# Pathological changes within the cerebral vasculature in Alzheimer’s disease: New perspectives

**DOI:** 10.1111/bpa.13061

**Published:** 2022-03-14

**Authors:** Robert A. Fisher, J. Scott Miners, Seth Love

**Affiliations:** ^1^ Dementia Research Group University of Bristol Medical School Bristol UK

**Keywords:** Alzheimer's disease, blood‐brain barrier, cerebral blood flow, neurovascular coupling, pericyte, vasculature

## Abstract

Cerebrovascular disease underpins vascular dementia (VaD), but structural and functional changes to the cerebral vasculature contribute to disease pathology and cognitive decline in Alzheimer's disease (AD). In this review, we discuss the contribution of cerebral amyloid angiopathy and non‐amyloid small vessel disease in AD, and the accompanying changes to the density, maintenance and remodelling of vessels (including alterations to the composition and function of the cerebrovascular basement membrane). We consider how abnormalities of the constituent cells of the neurovascular unit – particularly of endothelial cells and pericytes – and impairment of the blood‐brain barrier (BBB) impact on the pathogenesis of AD. We also discuss how changes to the cerebral vasculature are likely to impair Aβ clearance – both intra‐periarteriolar drainage (IPAD) and transport of Aβ peptides across the BBB, and how impaired neurovascular coupling and reduced blood flow in relation to metabolic demand increase amyloidogenic processing of APP and the production of Aβ. We review the vasoactive properties of Aβ peptides themselves, and the probable bi‐directional relationship between vascular dysfunction and Aβ accumulation in AD. Lastly, we discuss recent methodological advances in transcriptomics and imaging that have provided novel insights into vascular changes in AD, and recent advances in assessment of the retina that allow in vivo detection of vascular changes in the early stages of AD.

## Graphical abstract

Structural and functional changes to blood vessels not only underpin disease pathogenesis in vascular dementia but are also major contributors to cognitive decline and disease progression in Alzheimer’s disease. We provide an updated review on the clinical and neuropathological vascular features (SVD, CAA) and underpinning functional changes to blood vessels in relation of disease pathology in Alzheimer’s disease. We also discuss recent methodological advances within the field that have provided novel insights into vascular changes in AD, including transcriptomics, imaging, and retinal assessment providing a window of opportunity for future clinical intervention.

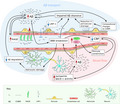

## 1 CEREBROVASCULAR DISEASE AND ALZHEIMER'S DISEASE

**FIGURE 1:**
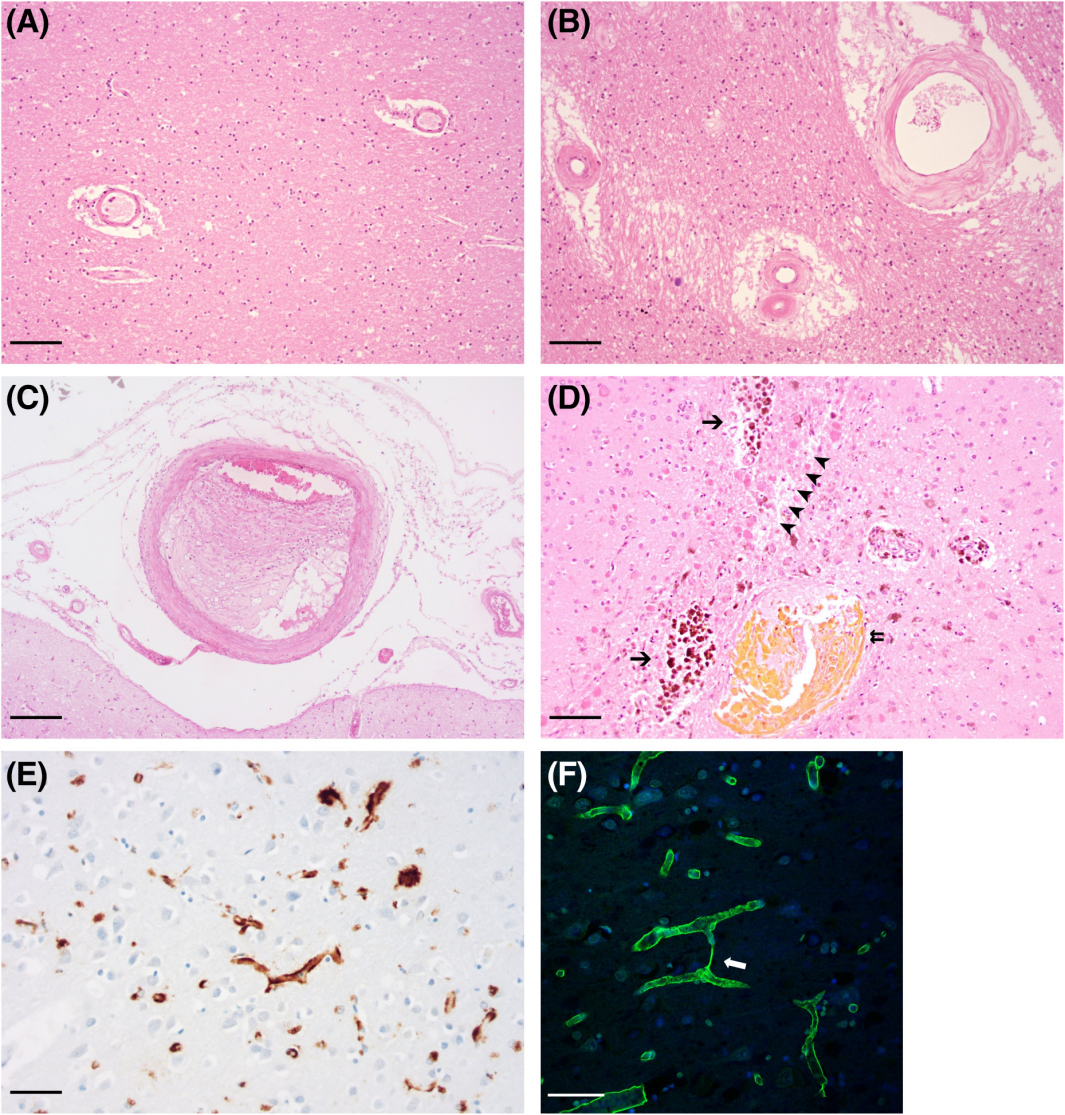
Common vasculopathies in AD. (A–D) Haematoxylin and eosin staining of fixed brain tissue sections with (A) normal white matter arterioles; (B) arterioles and artery with severe sclerosis; (C) artery with microatheroma, (D); non‐amyloid SVD‐associated microhaemorrhages – old (arrows) and more recent (double arrow) and a microinfarct (arrowheads). (E) Immunoperoxidase labelling of Aβ revealing Type‐2 CAA. (F) Immunofluorescent labelling of collagen (green) with DAPI (blue) nuclear stain reveals a string vessel (white arrow) connecting adjacent capillaries. Scale bars: (A) 100 µm (B) 100 µm (C) 200 µm (D) 100 µm (E) 50 µm (F) 50 µm.

Alzheimer's disease (AD) and vascular dementia (VaD) account for approximately 60%–80% and 5%–10% of patients with dementia, based on clinical [1] and neuropathologic diagnosis [2]. Most patients, including the majority with AD, have mixed pathologies that include pathological evidence of cerebrovascular disease [3, 4, 5]. Ischaemic damage to the white matter, attributed to small vessel disease (SVD), is associated with an increased risk of developing AD [6], and over 90% of AD patients have cerebral amyloid angiopathy (CAA; Figure 1E) [7, 8, 9]. In VaD, cerebral ischaemia is the defining pathological process, usually secondary to non‐amyloid, arteriolosclerotic small vessel disease (NA‐SVD; Figure 1A‐B) [10, 11], though also linked to ischaemic stroke injury [12]. There is also evidence of NA‐SVD comorbidities in AD cases [13, 14, 15] and the presence of NA‐SVD may be a risk factor for the development of AD [16].

A large scale multifactorial analysis of brain images from the Alzheimer's Disease Neuroimaging Initiative (ADNI) and modelling of late‐onset AD (LOAD) suggest that vascular dysregulation is an early and possibly an initial pathological event in AD [17, 18]. Cerebral hypoperfusion [19, 20] and blood‐brain barrier (BBB) breakdown [21, 22] precede the clinical presentation of dementia. Though BBB breakdown does occur in normal ageing, it is exacerbated in the early stages of AD, particularly within the hippocampus [21], and is associated with cognitive decline independently of changes in Aβ and Tau [23, 24]. In familial AD, reduction in cerebral blood flow (CBF) and glucose uptake occur soon after initial Aβ deposition and well before clinical disease [25, 26, 27]. There is also increasing evidence that malfunction of the neurovascular unit, partly related to injury to pericytes, is an early contributor to the development of AD [21, 28, 29].

**FIGURE 2:**
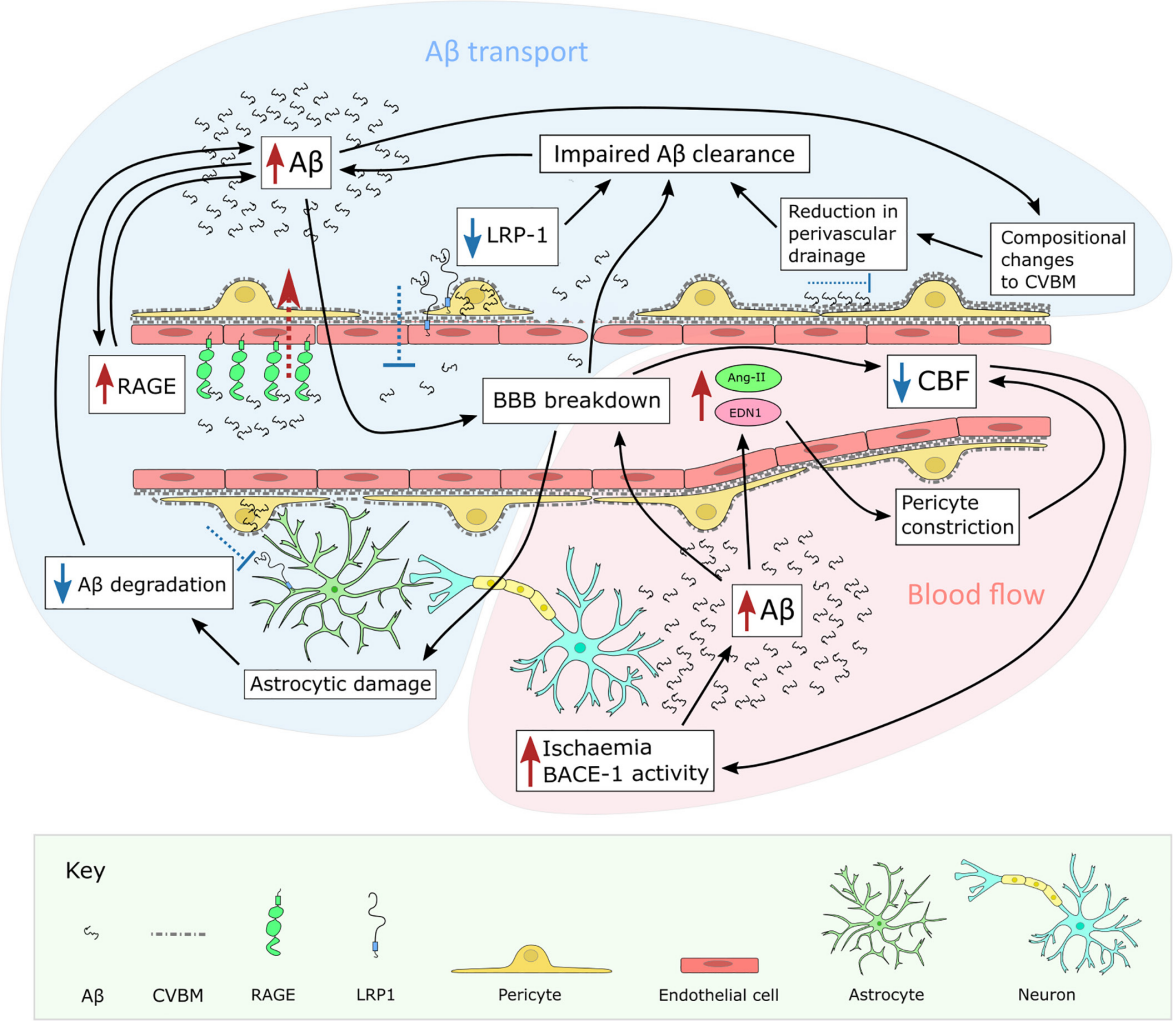
Vascular dysfunction and Aβ homeostasis. Aβ transport. Depletion of receptors involved in the transport of Aβ out of the brain parenchyma such as LRP‐1, upregulation of RAGE which transports Aβ into the parenchyma, impaired perivascular drainage, reduction of Aβ degradation, and BBB breakdown all contribute to Aβ accumulation in the brain in AD. Increased Aβ exacerbates BBB breakdown and CVBM alterations, constituting a vicious pathological cycle. Blood flow. Ischaemia elevates BACE‐1 activity which increases the processing of APP to Aβ, leading to oxidative stress, and upregulation of the vasoconstrictors angiotensin‐II (Ang‐II) and endothelin‐1 (EDN1). These stimulate pericyte constriction, lowering blood flow and exacerbating ischaemia. Similar pathological cycles may exist not only for Aβ but for other proteins associated with AD such as tau and TDP‐43

Pathological changes to the cerebral vasculature influence several processes involved in the progression of AD. Damage to the vasculature impairs periarteriolar clearance of Aβ (reviewed here Refs. [30, 31, 32, 33]) and receptor‐mediated removal of Aβ peptides across the BBB (reviewed here Refs. [34, 35, 36, 37]), accelerating Aβ deposition. Reduced cerebral oxygenation as a result of diminished blood flow and impaired neurovascular coupling enhance amyloidogenic processing of APP (Figure 2; reviews of in vitro and in vivo evidence here [5, 38]). Aβ peptides themselves are vasoactive, inducing contraction of pericytes [39] and vascular smooth muscle cells [40] which exacerbates hypoperfusion [39], and impairs BBB function [41]. Strong relationships have also been reported between measures of vascular dysfunction and the accumulation of phospho‐tau [42, 43, 44, 45] and TDP‐43 [11, 46] indicating that vascular dysfunction extends beyond Aβ pathology. Several therapeutic interventions have been proposed for the prevention or treatment of AD through improving cerebral hypoperfusion; these include the administration of vascular growth factors (reviewed here Ref. [47]).

Here we review the literature and provide an update on the structural, morphological, and functional changes to the cerebral vasculature in AD and discuss novel methods for investigating the cerebral vasculature in AD including examination of the retina.

## 2 COMMON VASCULAR PATHOLOGIES IN AD: CEREBRAL AMYLOID ANGIOPATHY, AND NON‐AMYLOID SMALL VESSEL DISEASE

Cerebral small vessel disease (SVD) is a major contributor to cognitive impairment in VaD (including post‐stroke dementia), and a frequent co‐pathology in AD [6, 10, 48, 49, 50, 51, 52, 53]. Neuroimaging features of SVD include white matter hyperintensities, infarcts, lacunae, haemorrhage, microbleeds and enlarged perivascular spaces – commonly reported in VaD and AD [52, 54, 55, 56, 57]. There are several types of SVD, the two most common being cerebral amyloid angiopathy (CAA) and non‐amyloid SVD (NA‐SVD) [51, 52].

### 2.1 Cerebral amyloid angiopathy

Aβ deposits not only in the brain parenchyma as plaques, but also in the walls of blood vessels, particularly arterioles, causing CAA. CAA affects 30%–40% of elderly people without dementia, the proportion increasing with age from about 65 years [58, 59], and over 80% of people with AD [7, 8, 9, 60, 61 ]. CAA is associated with increased risk of dementia, and more rapid cognitive decline in AD [62]. The severity of CAA has been assessed using a validated semiquantitative protocol [63] or by counting the number of neuroanatomical regions affected by CAA [64]. Arteriolar CAA and capCAA are most prevalent in the occipital lobe (>92% and 35%–47% of AD cases; 86% and 21% of non‐AD controls [63]).

*APOE* influences the risk of developing sporadic CAA [56, 65]. The severity of arteriolar CAA is increased by possession of *APOE* ε4 [7, 8, 9, 66, 67] which is also strongly associated with capillary CAA [63, 68, 69]. CAA is less prevalent in *APOE* ε2 suggestive of protection [63, 68, 69].

Sporadic CAA has been subdivided into two types. CAA‐Type 1 is more commonly associated with *APOE* ε4 and is characterised by Aβ deposition in cortical capillaries (capCAA) in addition to larger cortical and leptomeningeal vessels. In CAA‐Type 2, associated less strongly with *APOE* ε4 but also with ε2, Aβ accumulates in arteries, arterioles, veins and venules but not capillaries [63, 68, 70, 71] (Figure 1E). Parenchymal plaques consist of Aβ isoforms that tend to terminate at the 42^nd^ amino acid, often modified with pyroglutamate [72]. Arteriolar deposits in CAA are mostly shorter Aβ_1‐40_ and Aβ_2‐40_ [72, 73, 74, 75, 76], though Aβ_1‐42_ predominates in capillary CAA [72, 77, 78]. Vascular Aβ peptides are likely to be of neuronal origin, and their deposition in the walls of blood vessels is probably promoted by impaired perivascular clearance (see below).

CAA can be familial or sporadic. Familial CAA is rare but often more severe, manifesting clinically at an earlier age than sporadic CAA [79]. Familial Aβ‐CAA is associated with APP mutations or duplications, or mutations in PSEN1 or PSEN2 [56]; several APP‐linked mutations are used in transgenic animals to model CAA (reviewed here Refs. [80, 81]). Rarer forms of familial CAA are caused by vascular deposition of other amyloid proteins, including cystatin C, transthyretin, gelsolin, prion protein and BRI2‐gene products [82].

Arteriolar deposition of Aβ commences in the extracellular matrix of the tunica media but may progress to replace all SMCs and other tissue elements within the vessel wall [83]. Several additional vasculopathic abnormalities may complicate CAA, including fibrinoid necrosis, microaneurysms, concentric splitting of the vessel wall, and hyaline vessel wall thickening and arteriolar degeneration, and formation of glomeruloid capillary clusters [55, 63, 84, 85]. These vasculopathies likely underlie the cerebrovascular dysfunction found in CAA, several of which have been directly associated with microbleeds and haemorrhage [84, 85, 86, 87, 88]. *APOE* ε2 is associated with both fibrinoid necrosis and CAA‐associated haemorrhage [89]. Another rarer but increasingly recognised vasculopathy is CAA‐related inflammation (CAA‐RI), which can be subtyped into inflammatory CAA (ICAA) and Aβ‐related angiitis (ABRA), often treatable with immunosuppressive therapy (reviewed here Refs. [90, 91]). Clinicoradiological criteria allow diagnosis of probable CAA‐RI, avoiding the need for biopsy in most cases [92].

A recent in vivo imaging study of APP/PS1 mice with CAA found microhaemorrhages mostly at vessel bifurcations or bends without amyloid deposition [93]. The authors posited that altered flow dynamics within CAA‐affected vessels cause blood leakage. In capCAA, Aβ accumulation in the vascular basement membrane leads to degeneration of endothelial cells, loss of TJs and BBB breakdown [94], often associated with severe AD pathology [71, 78]. As sometimes occurs in severe “dyshoric” arteriolar CAA [9], Aβ deposits in capCAA tend to extend into the adjacent brain parenchyma where they are associated with neuroinflammation and hyperphosphorylated tau [68, 95, 96].

CAA increases the risk of cerebral haemorrhage and infarction [9, 56]. Common clinical MRI imaging features include lobar intracerebral haemorrhage, microhaemorrhages, siderosis, and white matter hyperintensities (WMHs) [56, 88, 97, 98, 99 ]. CAA progressively decreases vascular reactivity [100, 101, 102, 103], increasing the probability of hypoperfusion and ischaemic brain damage. CAA is associated with cortical atrophy independent of AD [104]. Functional brain connectivity was shown to be attenuated in Dutch type hereditary CAA [105].

Sporadic CAA probably results from impaired clearance of perivascular Aβ from the brain [106, 107]. Diminished intra‐mural periarterial drainage (IPAD) and perivascular CSF influx [6, 83, 106, 108, 109, 110], endothelial transport across the BBB [68, 70, 111, 112], or enzymatic degradation of Aβ [113, 114] may all contribute. By impairing vasomotion, Aβ‐mediated dysfunction of vascular smooth muscle may impede intramural clearance of Aβ within the interstitial fluid [115].

### 2.2 Non‐amyloid small vessel disease

NA‐SVD, sometimes referred to as ‘hypertensive angiopathy’, typically affects the small perforating arteries of the deep grey and white matter [51, 52]. Half of VaD cases are preceded by mild vascular cognitive impairment, which is also associated with NA‐SVD [15, 116, 117]. There is debate as to whether NA‐SVD may increase the likelihood of developing AD [16]. Age, smoking, diabetes and hypertension are risk factors for NA‐SVD [5, 15, 51, 52]. The genetic risk factors for NA‐SVD are still unclear but variation at several gene loci has been associated with its typical clinical and radiological manifestations [118]. We proposed previously that hypertension could be not only a cause but also a consequence of NA‐SVD: a cardiovascular response that is induced to maintain cerebral perfusion in the face of increasing cerebral vascular resistance [15]. This presents the possibility of a vicious cycle between hypertension and NA‐SVD, a possibility supported by our recent demonstration that although late‐life hypertension was associated with markers of vascular damage (SVD severity and BBB breakdown), it was also associated with evidence of better cerebral perfusion and lower insoluble Aβ42 levels in AD and mixed dementia [119].

The core pathology of NA‐SVD is arteriolosclerosis (Figure 1B): collagenous thickening of the vessel wall, narrowing of the vessel lumen and loss of SMCs. NA‐SVD can be further categorised by the presence or absence of distal atherosclerosis (microatheroma; Figure 1C), lipohyalinosis, fibrinoid necrosis or microaneurysms [11, 51, 52, 120, 121, 122, 123, 124, 125 ]. Histopathological measures of NA‐SVD usually rely on semiquantiative scoring of arteriolosclerosis, for example using the criteria in the Vascular Cognitive Impairment Neuropathology Guidelines (VCING). Another metric, the sclerotic index, also been used as a measure of NA‐SVD pathology in brain tissue [126, 127, 128, 129].

We have previously scored the severity of NA‐SVD pathology in tissue sections using a semiquantitative scale that was incorporated into VCING and based on the extent of thickening of the arteriolar walls and narrowing of the lumens [119, 130, 131]. We showed that individuals with severe NA‐SVD had reduced levels of vWF in the white matter, indicative of vessel loss [15, 130]. Perivascular drainage is likely to be impaired (as suggested by the enlarged perivascular spaces [52, 132, 133]). Like CAA, NA‐SVD is associated with lacunar infarcts, haemorrhage and microbleeds but their distribution varies between these two forms of SVD [33, 52, 134, 135] (Figure 1D). Microbleeds tend to be lobar in CAA, and non‐lobar (basal ganglia, internal capsule, thalami) in NA‐SVD [52]. Microbleeds are demonstrable in up to one‐third of AD patients [15, 136, 137, 138 ], and mostly lobar in distribution; however a substantial number are also non‐lobar and associated with WMHs, reflecting the high prevalence of both major types of SVD in AD [13, 14, 15]. It seems likely NA‐SVD reduces cerebrovascular reactivity but supporting data is scarce [33, 139]. Independently of cerebral infarction and CAA, arteriolosclerosis has been linked with limbic predominant age‐related TDP‐43 pathology [11, 46, 140, 141, 142, 143, 144 ], a proteinopathy that often coexists with AD [144, 145].

## 3 VASCULAR DENSITY AND REMODELLING IN AD

### 3.1 Changes in vascular density

Most studies on human brain tissue have reported reduced or no significant changes in vascular density (Table 1). Reported reductions in vascular density have tended to be region‐specific and related to disease progression and the presence of disease pathology (Table 1). A reduction in retinal vascular density has also been reported in AD (see section 7 and Table 2). In transgenic mouse and rat models of Aβ accumulation, vascular density is generally decreased (Table 2), though two recent studies reported a transient increase in vascularity, mostly hippocampal, in early disease but a subsequent decline in vascular density with disease progression [146, 147]. This may indicate an early stage angiogenic response that is ultimately ineffective as disease progresses. A few human post‐mortem studies found increased vascular density in AD within the hippocampus [148, 149, 150] (Table 1) and the frontal cortex [151], though several other studies found either no significant change or a reduction in vascularity in these regions (Table 1).

**TABLE 1:** Vascular density reported in human patients with AD

Author/year	Change in VD	Region	Method; metric	Stage of disease progression
Fernandez‐Klett et al. (2020) [151]	Increase	Frontal cortex	IHC (CD31); vessel length density	AD
Burke et al. (2014) [150]	Increase	Hippocampus	IHC (Glut1, ColIV, αSMA); vessel length density	AD
Desai et al. (2009) [149]	Increase in hippocampus	Multiple regions	Bright field microscopy; branch counts	AD
Meier‐Ruge et al. (1985) [154]	Increase in capillary volume and length	Multiple regions	Alkaline phosphatase lead‐staining; multiple metrics	AD
Bell et al. (1981) [148]	Increase	Hippocampus	Alkaline phosphatase lead‐staining; multiple metrics	AD
Bell et al. (1986) [354]	NS change	Hippocampus and calcarine (visual) cortex	Alkaline phosphatase lead‐staining; vessel length density	AD
Kawai et al. (1990) [355]	NS change	Hippocampus	IHC (GLUT1 & ColIV labelling); vessel counts	AD
Bell et al. (1990) [356]	NS change	Visual cortex	Alkaline phosphatase lead‐staining; vessel length density	AD
Hunter et al. (2012) [155]	NS change	Multiple regions	IHC (ColIV); multiple metrics	AD
Harris et al. (2018) [357]	NS change	Parietal cortex	ELISA (vWF); vWF mU/mL	AD
Miners et al. (2017) [277]	NS change	Precuneus	Dot blot (vWF); dot intensity	AD
Thomas et al. (2015) [358]	NS change	Multiple regions	Dot blot (vWF); dot intensity	AD
Kirabali et al. (2020) [156]	NS change	Cortex and hippocampus	IF (fluorescent lectin); multiple metrics	AD
Ding et al. (2020) [276]	NS change	Frontal lobe white matter	IHC and IF (ColIV); vessel length density	AD
Ding et al. (2021) (285)	NS change	Frontal cortex	IHC and IF (ColIV); vessel length density	AD
Damodarasamy et al. (2020) [206]	NS change	Frontal and parietal cortices	IHC (CD31 and vWF); vessel area density	AD
Fischer et al. (1990) [153]	Decrease	Multiple regions	Alkaline phosphatase lead‐staining; branch counts	AD
Brown et al. (2007) [359]	Decrease	White matter	Alkaline phosphatase lead‐staining; vessel area density	AD
Lepelletier et al. (2017) [186]	Decrease	Multiple regions	IHC (vWF); vessel area density	Preclinical AD & AD
Buee et al. (1994) [360]	Decrease	Cortex	IHC (HSPG); vessel area density	AD
Baloyannis et al. (2012) [161]	Decrease	Multiple regions	Golgi silver staining and electron microscopy; branch counts	AD
Kitaguchi et al. (2007) [157]	Decrease	Cortex	Gallyas silver staining; branch counts	AD
van de Kreeke et al. (2019) [332]	Increase	Retina	OCTA	Preclinical AD (Aβ+)
den Haan et al. (2019) [329]	NS or no change	Retina	OCTA	AD
Zhang et al. (2019) [319]	Decrease	Retina	OCTA; vessel length density	Early AD and aMCI
Yoon et al. (2019) [320]	Decrease with AD, not with MCI	Retina	OCTA	MCI & AD
Jiang et al. (2018) [321]	Decrease	Retina	OCTA; box counting	MCI & AD
Grewal et al. (2018) [322]	Decrease	Retina	OCTA	AD
Bulut et al. (2018) [323]	Decrease	Retina	OCTA	AD
Yan et al. (2021) [324]	Decrease	Retina	OCTA	AD
Wang et al. (2021) [325]	Decrease	Retina	OCTA	MCI & AD
Chua et al. (2020) [326]	Decrease	Retina	OCTA	MCI & AD
Wu et al. (2020) [327]	Decrease	Retina	OCTA	MCI & AD
Shin et al. (2021) [331]	Decreased; worse in APOE ε4 carriers	Retina	OCTA	MCI

Abbreviations: AD, Alzheimer's disease; IF, immunofluorescence; IHC, immunohistochemistry; MCI, Mild cognitive impairment; mo, months; NS, no significant; OCTA, optical coherence tomography angiopathy; VD, vascular density.

**TABLE 2:** Vascular density reported in AD‐like animal models

Author/year	Change in VD	Animal model	Region	Method; metric	Age
Bennett et al. (2018) [42]	Increase	Mouse (P301L)	Cortex	In vivo two‐photon imaging of fluorescein‐conjugated dextran perfused brain; vessel volume density, vessel length density	2, 9, 12, 15 and 18 mo
Delafontaine‐Martel et al. (2018) [349]	NS or no change	Mouse (APP/PS1)	Whole brain	Two‐photon imaging of FITC‐gelatin perfused brain; vessel volume density	2, 4. 5 and 8 mo
Nikolajsen et al. (2015) [361]	NS or no change	Mouse (APPswe/PS1dE9)	Multiple regions	IHC (CD31); vessel length density	18 mo
Paris et al. (2004a) [362]	Decrease	Mouse (Tg4510)	Hippocampus; cyngulate cortex and neocortex	IHC (PECAM‐1); vessel counts	9 mo
Herring et al. (2016) [363]	Decrease	Mouse (TgCRND8)	Neocortex and basal ganglia	IHC (laminin); branch counts	360 d
Zhang et al. (2019) [364]	Decrease	Mouse (3xTg‐AD)	Neocortex and underlying collateral zone	IF (GLUT‐1); semi‐quantitative scoring	8 and 18 mo
Miao et al. (2005) [365]	Decrease	Mouse (Tg‐SwDI)	Cortex, hippocampus, and thalamus	IHC (Collagen IV); vessel counts	3–24 mo
Keyvani et al. (2018) [366]	Decrease	Mouse (TgCRND8)	Cortex, hippocampus, and thalamus	IHC (Laminin); vessel area density	90, 180 & 360 d
Lee et al. (2005) [367]	Decrease	Mouse (dtg‐APP/PS1)	Corpus callosum	IHC (β‐NADPH); multiple metrics	7 mo
Li et al. (2019) [162]	Decrease	Mouse (PS1 / tauP301L / APPSwe)	Whole brain	HFμDI; vessel volume density	4 and 11 mo
Zhang et al. (2019) [165]	Decrease	Mouse (APP/PS1)	Whole brain	IF (Nissl); multiple metrics	2–24 mo
Kouznetsova et al. (2006) [368]	Decrease	Mouse (Tg2576)	Cortex	IF (Glut1); semi‐quantitative scoring and % capillary load	4–18 mo
Giuliani et al. (2019) [146]	Decrease^a^	Mouse (Tg2576)	Cortex	IF (laminin); vessel volume density	1–27 mo
Xu et al. (2020) [147]	Decrease^a^	Mouse (APP23)	Cortex and hippocampus	MRI; multiple metrics	3–20 mo
Stefanova et al. (2018) [369]	Decrease	Rat (OXYS)	Hippocampus	Bright field microscopy; vessel counts	20 d, 5 mo, 18 mo

Abbreviations: d, days; H&E, haematoxylin and eosin; HFμDI, high‐frequency micro‐Doppler imaging; IF, immunofluorescence; IHC, immunohistochemistry; mo, months; tg, transgenic; VD, vascular density.

^a^ Transient increase in VD observed in young mice.

Advanced age is associated with a decline in vascular density (reviewed here Ref. [152]), making it important to use age‐match controls when assessing vascular changes in the context of AD. The reduced vascular density in AD reported by Fischer and colleagues (1990) may have been confounded by the younger age of the controls (mean 60.8 years) compared to AD cases (mean 84.8 years) [153]. Advanced age is also associated with cerebral atrophy, but in most brain regions this is more marked in AD. Brain atrophy may reduce separation of blood vessels, increasing vascular density without the generation of new vessels. Meier‐Ruge and colleagues (1985) attributed an increase in vascular density in the AD brains they studied to tissue atrophy [154]. However, Hunter and colleagues (2012) reported no change in vascular density in AD, despite tissue atrophy [155], and Kirabali and colleagues (2020) found no difference in vascular density in the frontal cortex and hippocampus in AD despite a reduction in the nearest distance between capillaries, perhaps reflecting parenchymal atrophy [156]. Kitaguchi et al. (2007) observed lower vascular density in AD despite greater atrophy than in controls [157].

Discrepant data on vascular density in AD probably reflect differing methods, metrics, brain regions, disease stage, and confounders such as comorbidity or age. The heterogeneous distribution of tissue atrophy across the brain and between individuals [158, 159, 160] further complicates comparison of vascular density measurements in AD.

### 3.2 Morphological changes in the vasculature

Numerous morphological abnormalities have been reported in AD and animals modelling aspects of the disease. These include increased vascular tortuosity and looping and kinking in AD [153, 161] and transgenic mice overexpressing APP [162, 163]; irregularities in capillary diameter in AD [157, 161, 164] and APP/PS1 mice [165]; abnormal patterns of branching, fusion and budding of vessels in AD [161] and APP23 mice [157, 163]; and an increase in degenerated ‘string’ vessels in AD [155, 166]. Raspberry‐like clusters of cerebrocortical microvessels that probably reflect an angiogenic response to brain ischaemia were found to be most numerous in VaD but also more numerous in frontotemporal lobar degeneration and AD than in controls [167].

Accumulation of tau may also contribute to vascular abnormalities. Overexpression of tau in mice caused a range of vessel abnormalities including reductions in diameter, the formation of vascular spirals, and altered vascular density [42].

### 3.3 Non‐productive angiogenesis in AD

The causes of most of the above changes to vascular morphology in AD remain unclear. Brain atrophy is likely to have deformed the morphology of some vessels [168]; however looping, budding, fusion and tapering of vessels may be secondary to angiogenic stimulation [163, 169]. String vessels in AD have been suggested to reflect a cycle of pathological angiogenesis and subsequent endothelial retraction [170] (Figure 1F). Recently reported ‘raspberries’ likely form through angiogenesis in response to tissue hypoxia [167].

Despite evidence of pro‐angiogenic signalling in AD, there is scant evidence of increased vascularization, possibly owing to anti‐angiogenic properties of Aβ [5, 171, 172]. In AD and animal models of Aβ accumulation, non‐productive angiogenesis was shown near amyloid plaques, with an abnormal accumulation of IB4‐positive tip cells and reduced NOTCH signalling. The angiogenic vessels were disassembled by microglial phagocytosis. Non‐productive angiogenesis also occurred in the absence of plaques in mice with inhibited γ‐secretase activity [173].

## 4 ALTERATIONS IN THE CEREBRAL VASCULAR BASEMENT MEMBRANE IN AD

The ECM provides structural stabilisation to the neurovascular unit by binding cell‐adhesion molecules. It also supports cell migration and differentiation, and facilitates cell signalling [174]. The cerebral vascular basement membrane (CVBM) is a specialised ECM composed of laminin, collagen IV, nidogen, heparan sulphate and proteoglycans. It encloses endothelial cells and pericytes and supports interactions between them, and with astrocytes through their endfeet [175]. Changes to the CVBM in AD have adverse consequences for vascular function and Aβ efflux (reviewed here Refs. [31, 174, 175, 176, 177, 178]). The major alterations to the CVBM in AD include thickening, changes in composition, and Aβ deposition [175].

### 4.1 CVBM thickening

The CVBM thickens with age, but this process is exacerbated in AD and in animal models of Aβ accumulation [168, 179, 180, 181, 182, 183, 184, 185, 186], tending to be most severe in regions of the brain with higher levels of AD pathology [180, 186]. It is attributable mostly to increased collagen IV [94, 182, 183, 187, 188, 189], although perlecan and fibronectin are also increased [186, 190]. One study found elevated collagen I and III but reduced collagen IV [191]. A few reports describe CVBM thinning in AD microvessels, though those studies focussed on agrin and may reflect decreases in that particular component [192, 193, 194]. Thickening increases the stiffness of the BM, and probably attenuates vascular compliance and neurovascular coupling. Reduced contact between endothelial cells and pericytes [195] may also affect BBB function and the stability of capillaries as a result of CVBM thickening.

### 4.2 CVBM compositional changes

In addition to the deposition of collagen, increases in perlecan and fibronectin were found in AD microvessels [186] (although not in *APOE* ε4 mice [196]). These constituents contribute to maintaining the endothelial barrier and mediating cell attachment and function [197, 198]. Plasma fibronectin was increased in AD but reduced in MCI [199, 200]. Laminin α1, β1 and γ1 expression was increased in brain tissue and astrocytes in AD [201, 202] and small laminin peptides were elevated in the CSF [203]. Laminin and other components of the CVBM, including collagen IV and heparan sulphate proteoglycan, also colocalise with amyloid plaques, which reflect CVBM damage [204, 205]. However, both collagen IV and laminin were reportedly reduced in microvessels from AD brain tissue, while laminin was elevated in microvessels with CAA. The same study found that levels of perlecan and fibronectin were unchanged in AD and CAA [206].

Intramural perivascular drainage (IPAD) of interstitial fluid in the brain takes place along the vascular CVBM, particularly those that define the concentric layers of smooth muscle cells and is an important mechanism of Aβ clearance [207, 208]. Pathological remodelling of the CVBM can interfere with this process (Figure 2). Reduced compliance of vessels with thickened CVBMs in normal ageing may explain reduced cardiovascular pulse propagation [209], thought to drive perivascular transport in the brain via its reflection wave [31, 210, 211]. Additionally, elevated levels of perlecan, and fibronectin, owing to normal aging may facilitate the aggregation of soluble Aβ [180]. Additionally, CVBM laminin, which is reduced in ageing and CAA, binds APOE‐Aβ complexes and assists in the efflux of Aβ from the brain. This binding is weaker for APOE ε4‐Aβ than APOE ε3‐Aβ complexes [212]. Astrocytes from ε4‐positive individuals secreted less laminin and collagen IV, and more fibronectin when forming the CVBM [213]. Conversely, ECM laminin and collagen were increased in ε2 carriers [214]. These differences in composition of the CVBM may influence the efficiency of intramural periarterial drainage. Changes in CVBM and ECM composition can also affect Aβ fibrillation and stability. For instance, collagen IV, laminin and nidogen can disrupt the formation of Aβ fibrils [215, 216]. Conversely, proteoglycans such as perlecan and agrin aid Aβ fibril formation and stability [217, 218, 219].

Caution is warranted in interpreting some of the data on immunolabelling components of the CVBM, which is highly cross‐linked. The detection of CVBM components is affected by variability in antigen affinity and accessibility (e.g. in a study on formalin‐fixed tissue, laminin antibodies labelled neurons but not the CVBM unless extensively damaged [220]), in addition to autofluorescence [221].

## 5 THE NEUROVASCULAR UNIT IN AD

**FIGURE 3:**
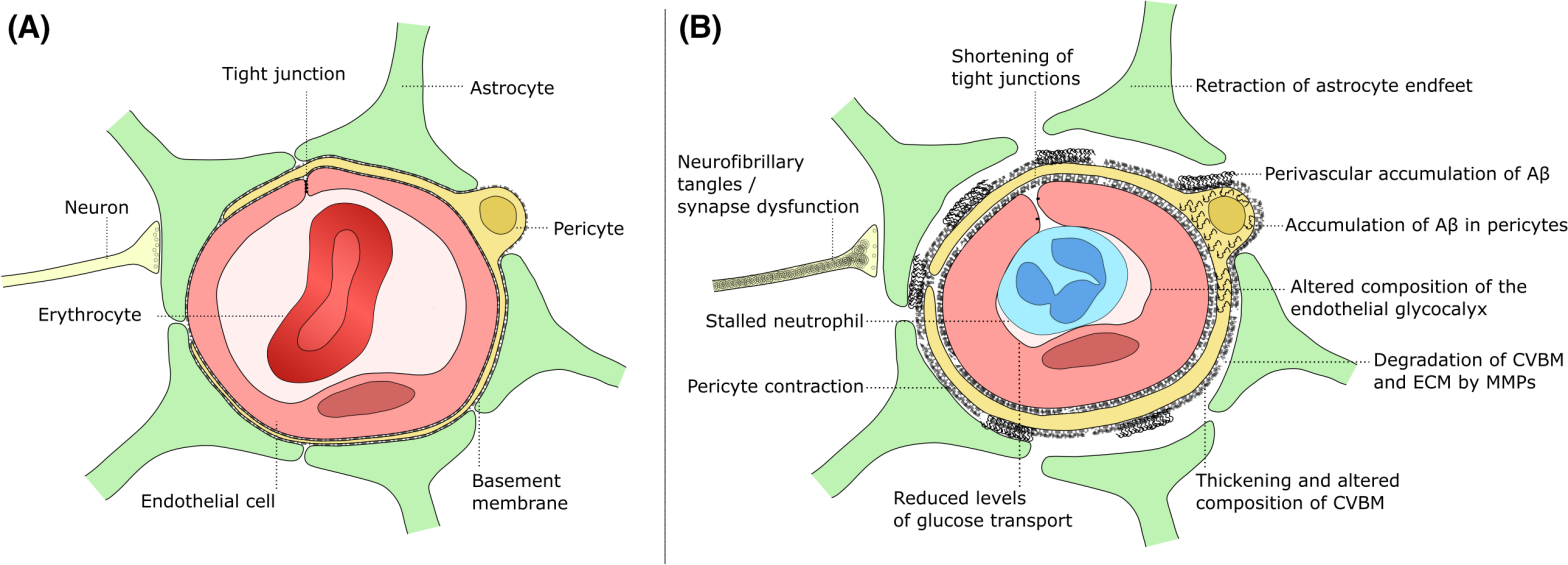
Structure of the microvascular NVU in health and AD. (A) Structure of NVU in the microvasculature. Endothelial cells form tight junctions that limit the transport of blood constituents. The tight junctions are a major component of the BBB, the integrity of which is further enforced by the basement membrane and pericytes surrounding the endothelium. Astrocytic endfeet cover most of the surface of the microvessel, and form water channels important for glymphatic transport and homeostasis within the brain. Neurons and astrocytes can signal to pericytes to modulate their state of constriction, linking regional CBF to neuronal activity. (B) NVU pathology and dysfunction in AD. Numerous changes in the function and physiology of the NVU have been described in the context of AD (outlined here), many of which are also seen to a lesser extent in normal aging

Endothelial cells, mural cells, astrocytes and neurons work in concert in the healthy brain to form the neurovascular unit (NVU), which regulates neurovascular coupling and consequently CBF through the brain (reviewed here Refs. [222, 223]). The CVBM and endothelial tight junctions are important non‐cellular structures of the BBB, which sits within the NVU (Figure 3). NVU dysfunction and BBB barrier disruption in AD are closely associated with one another [223]. Below, we describe various pathologies and dysfunction with a focus on the vascular components of the NVU in AD. Glial cells are also important in the normal function of the NVU and contribute to its disruption in AD. Astrocytes are important for glymphatic drainage of Aβ [224, 225] and microglial neuroinflammation has multiple effects on vascular function, as reviewed elsewhere [226, 227]. However, detailed consideration of the roles of astrocytes and microglia in AD is beyond the scope of the present review.

### 5.1 Endothelial cells

Reported abnormalities of endothelial cells in AD include mitochondrial damage, increased pinocytic vesicles and lipofuscin [161, 170]; upregulated receptor for advanced glycation end products (RAGE), responsible for transporting Aβ into the brain parenchyma [228, 229, 230]; and downregulated low density lipoprotein receptor‐related protein 1 (LRP‐1) receptor, involved in Aβ clearance into the bloodstream [228, 231] (Figure 2). Lower LRP‐1 levels may reflect diminished expression of MEOX2 [232].

Glucose transport across the endothelium and BBB is impaired in AD. Expression of glucose transporter proteins GLUT‐1 and GLUT‐3 is reduced in the endothelium of microvessels in the cerebral cortex, and hippocampus in AD [233, 234, 235, 236, 237], and GLUT‐1 levels are reduced in circulating brain endothelial cells in mild AD [238]. This may exacerbate Aβ and cerebrovascular pathology. A fall in GLUT‐1 and retraction of astrocytic endfeet preceded widespread Aβ pathology in arcAβ mice [184]. Knockdown of GLUT‐1 in zebrafish caused BBB breakdown and impaired cerebral circulation [239]. In mice over‐expressing APP, GLUT‐1 deficiency led to early cerebral microvascular degeneration, BBB breakdown, reduced CBF, impaired neurovascular coupling, accelerated Aβ accumulation and neurodegeneration [240].

Endothelial cells in AD patients and 5xFAD mice have shortened tight junctions (TJs) [161, 241], important for maintaining BBB integrity. Additionally, there is loss of TJ proteins occludin, claudin‐5 and ZO‐1 in CAA; in vitro experiments suggested that this results from Aβ toxicity to endothelial cells, mediated by the binding of Aβ to endothelial RAGE and induction of oxidative stress [242, 243]. Other studies showed an association of RAGE, Ca^2+^‐calcineurin signalling and MMP expression, with a decrease in TJ protein levels in endothelial cells [41, 241]. Hypoxia, hypoglycaemia and oxidative stress – conditions prevalent in AD brain tissue – all decrease TJ protein levels in endothelial cells in vitro [244, 245, 246]. Disruption of TJs in AD brains is associated with BBB breakdown and increased immune cell infiltration [247] and in Tg2576 mice, disrupted TJs were linked to angiogenesis [248].

Markers of endothelial activation, including intercellular adhesion molecule‐1 (ICAM‐1) and vascular adhesion molecule‐1 (VCAM‐1), have been reported to be elevated in AD and in animal models of disease [249, 250, 251, 252, 253], either as a result of inflammation or direct exposure to Aβ [253, 254]. This facilitates entry of immune cells into the brain parenchyma where they can exacerbate neuroinflammation and AD pathology [253]. Neutrophil depletion in 3xTg‐AD mice was shown to decrease AD pathology and improve memory [253]. Elevated expression of cell adhesion molecules may also contribute to neutrophil blockage of capillaries observed in APP/PS1 mice [255]. Treatment of these mice with antibody to the neutrophil marker Ly6G increased CBF, possibly by inhibiting the migration of neutrophils towards endothelial inflammation [255].

The glycocalyx on the luminal surface of the endothelium of the brain is an important component of the BBB that shows signs of damage in AD and CAA (reviewed here Ref. [177]). Levels of glycocalyx components hyaluronan and TSG‐6 were increased in microvessels from brain tissue with AD or CAA, which is characteristic of endothelial inflammation and injury [206].

### 5.2 Pericytes

Pericytes are recruited by endothelial cells in nascent microvessels by signalling between platelet‐derived growth factor‐BB (PDGF‐BB) and its cognate pericyte receptor PDGFRβ. Pericytes regulate an array of processes including BBB functioning, TJ formation, ECM remodelling, angiogenesis, metabolite clearance, and coordinate signalling between other cell types of the NVU [223, 256, 257, 258, 259, 260, 261, 262]. Mice deficient in PDGFRβ‐signalling experience early loss of pericytes, and BBB breakdown [263]. Pericytes can also clear Aβ aggregates via LRP1/ApoE [264] and regulate local CBF through constriction or relaxation of their processes, modulating capillary diameter [265, 266, 267]. Pericyte relaxation is stimulated by neuronal activity, positioning pericytes as important mediators of neurovascular coupling, the process in which CBF is linked to neuronal activity [261, 268, 269]. Embedded within the CVBM, pericytes are able to communicate with endothelial cells through small gaps in the CVBM where the plasma membranes of both cells come into contact [270]. Pericytes can differentiate into various components of the NVU following ischaemic damage [271, 272], suggesting an important role in tissue repair. However, ischaemia was also shown to cause pericyte death ‘in rigor’, irreversibly constricting microvessels and leading to BBB damage [261].

#### 5.2.1 Pericyte dysfunction and loss

Pericyte‐deficient mice manifest an age‐dependent decrease in brain perfusion and neurovascular coupling, associated with BBB breakdown, neurodegeneration and cognitive impairment [260]. These mice also display accelerated Aβ deposition when crossed with animal models of Aβ accumulation [29, 259, 273].

Reduction in pericyte coverage of capillaries was reported in neocortex and hippocampus from AD patients, correlating with BBB breakdown [29, 156, 274]. However, in some studies, capillary pericyte counts were stable in the frontal cortex in AD [151, 275]. This discrepancy could reflect a loss of pericyte processes rather than of pericytes themselves in AD. The number of pericytes was reduced in the frontal white matter in AD, VaD, post‐stroke dementia and mixed dementia [276]. We found a reduction in the pericyte marker PDGFRβ in the precuneus, a region hypoperfused early in AD; the reduction correlated with an increase in fibrinogen indicating BBB breakdown, and with hypoxia and Aβ plaque load [277]. We did not observe a reduction of PDGFRβ in white matter underlying the precuneus. Bourassa et al., measured mural cell markers – PDGFRβ, CD13 and α‐SMA, in microvessels extracted from the parietal cortex of 60 participants in the Religious Orders study and found the marker levels to be reduced in AD in association with TDP‐43 levels [278].

Electron microscopy of the hippocampus, visual, auditory and parietal cortices in AD revealed mitochondrial abnormalities and increased numbers of pinocytic vesicles in pericytes along with a reduction in their overall number; this coincided with a shortening of TJs [161]. In patients with MCI, injury to brain pericytes, assessed by measuring sPDGFRβ in the CSF, was associated with evidence of BBB breakdown on dynamic contrast‐enhanced MRI, and elevated CSF albumin [21, 24, 279]. Pericyte degeneration and BBB breakdown are accelerated in *APOE* ε4 carriers [280].

Pericytes accumulate Aβ in mouse models of AD, and Aβ_1‐40_ fibrils reduced pericyte viability and proliferation in vitro [264, 274] (interestingly, Aβ_1‐40_ monomers had the opposite effect on pericytes, suggesting that the effect of Aβ on pericyte function is aggregation‐dependent, as it is with endothelial cells [274]). Aβ oligomers induced constriction of capillaries by pericytes in human *ex vivo* tissue and animal models of AD, a process dependent on ROS generation and EDN1 [39]. Capillaries from AD patients were constricted specifically at pericyte locations, with no concomitant change in the diameters of distal arterioles or venules. Pericyte constriction may therefore be chiefly responsible for reduced CBF in AD [39]. BACE‐1 levels increase under hypoxia, owing to a hypoxia‐responsive element in the *BACE*‐*1* promoter, which leads to increased processing of APP into Aβ [281, 282]. This probably results in further pericyte constriction and reduced CBF, forming a vicious cycle [38] (Figure 2). We recently showed that exposure to Aβ interferes with EDN1‐mediated constriction and relaxation of pericytes in vitro [283], which would be expected to impair neurovascular coupling. It is also possible that this interference in the rhythmic constriction and relaxation of mural cells may reduce the effectiveness of clearance of Aβ through IPAD.

## 6 THE BLOOD‐BRAIN BARRIER IN AD

The structure and function of the BBB have been extensively reviewed [284, 285, 286, 287, 288], as has its disruption in AD [23, 289, 290, 291]. Damage of the BBB was detected preclinically in AD within the hippocampus, independently of the progression of Aβ and tau pathology [24, 289]. BBB breakdown occurs in animal models of Aβ (reviewed here Ref. [292]) and tau accumulation [293], and in AD patients [23, 170, 289]. Blood constituents, including thrombin, fibrinogen, IgG, albumin, and haemoglobin‐derived proteins, can be detected in the brain parenchyma in AD, often in association with amyloid plaques [29, 194, 277, 280, 294, 295, 296, 297]. Albumin levels tend to be elevated in the CSF in MCI and AD [21, 23, 277, 298, 299, 300, 301]. Within the brain parenchyma, plasmin, fibrinogen, thrombin, and albumin can cause neurovascular damage, inflammation, oedema, and ECM degradation [23]. The pathology resulting from plasmin leakage alone was demonstrated by the markedly reduced inflammation and Aβ deposition that followed depletion of plasminogen in Tg6799 mice [302].

An age‐dependent increase in BBB permeability correlates with a rise in the CSF level of a cleaved, soluble form of platelet‐derived growth factor receptor β (sPDGFRβ) shed from damaged pericytes; this is exacerbated in MCI [21]. We found that BBB breakdown (evidenced by accumulation of fibrinogen in post‐mortem tissue from individuals with AD, VaD and mixed dementia) was associated with increased endothelin‐1 (EDN1), more severe hypoperfusion (lower myelin‐associated glycoprotein:proteolipid‐1 ratio), SVD, Aβ and tau [119]. BBB breakdown in AD patients is associated with a reduction in CBF [23, 28, 303, 304, 305]. Tau‐PET imaging revealed a negative correlation between tau pathology and CBF in the temporoparietal regions, exacerbated by the presence of amyloid [43]. *APOE* ε4 carriers are at increased risk of early BBB breakdown and degeneration of pericytes [193, 194, 280, 297, 306].

Inappropriate activation of MMPs can cause BBB disruption (e.g. following cerebral ischaemia [307]). MMP‐9 knockout mice had reduced infarct volume, neurological deficits and mortality after focal cerebral ischaemia associated with the protection of the BBB [308]. Aβ‐induced activation of MMPs may damage the BBB in AD. In vitro, Aβ_1–42_ oligomers increased production of RAGE, MMP‐2, MMP‐9, and decreased levels of TJ proteins in bEnd.3 cells [41, 241]. The ensuing BBB disruption could be fully reversed by addition of an anti‐RAGE antibody and partially reversed by a general MMP inhibitor. Similarly, breakdown of the blood‐CSF barrier in mice after exposure to Aβ_1–42_ oligomers did not occur in the presence of MMP inhibitor or in MMP‐3‐deficient mice [309]. *APOE* ε4 also drives BBB breakdown, by activating the cyclophilin A‐MMP‐9 pathway in mice and non‐symptomatic human carriers [214, 260, 280, 298]. In vitro, pericytes produced MMP‐9 and migrated in response to TNF‐α [310] (a mediator of neuroinflammation in AD [311]); this could be blocked by anti‐MMP‐9 antibody [310].

Increased activity of MMPs may also contribute to non‐productive angiogenesis in AD (see above). Proteolysis of CVBM components by MMPs is necessary for endothelial migration and sprouting, and tube formation [312, 313, 314].

## 7 NOVEL INSIGHTS AND METHODS FOR STUDYING THE CEREBRAL VASCULATURE IN AD

### 7.1 Retinal studies

The study of the retina in AD research was recently reviewed by Shi et al. (2021) [315]. In a mouse model of Aβ accumulation, Shi and colleagues found vascular pathology and pericyte loss alongside retinal Aβ accumulation [316]. They also reported pericyte apoptosis and reduction in PDGFRβ and LRP‐1 associated with Aβ deposition in retinas examined post‐mortem from donors with MCI and AD, mirroring cerebral findings in AD [316]. Additionally, venular abnormalities, microglial activation and astrogliosis were recently demonstrated in the retina coinciding with accumulation of Aβ in an APP^NL‐G‐F^ knock‐in transgenic mouse model [317].

Optical coherence tomography angiography (OCTA) uses laser light reflectance off the surface of haemocytes in motion to produce a map of the microvessels in the retina [318]. Several OCTA studies found a decline in retinal vascular density in AD and MCI (Table 1), associated with morphological anomalies of the vasculature and an increased foveal avascular zone (FAZ) [319, 320, 321, 322, 323, 324, 325, 326, 327, 328]. Two studies reported no difference in retinal vascular density in AD [329, 330] although one did find a thinner choroid in mild AD [330]. Patients with MCI showed a decline in retinal vascular density [319, 321, 325, 326, 327, 331] (with one exception [320]), though to a lesser extent than in AD. Van der Kreeke et al. (2020) reported an increase in retinal vascular density in preclinical AD diagnosed by amyloid‐PET [332]. Changes in retinal vascular density, tortuosity, FAZ area and inner retinal layer thickness are being reported with increasing consistency in AD and other dementias [333, 334]. However, further studies, particularly in preclinical AD and MCI, and with correlative neuropathology, are needed to determine the diagnostic and prognostic value of OCTA in AD [335, 336].

### 7.2 Single‐cell transcriptomic studies of the cerebral vasculature

Single cell or nuclear RNA‐Seq (snRNA‐Seq) is a powerful tool for transcriptomic analysis of human tissue. Gene studies and snRNA‐Seq profiling have implicated microglia as having a central role in AD pathogenesis (reviewed here Refs. [337, 338, 339, 340]). Of recent snRNA‐Seq studies in AD [171, 341, 342, 343], few have focused on the cerebral vasculature, although recent findings include increased transcription of cytokines [343], angiogenic markers and proteins involved in endothelial antigen presentation in AD [171]. Two recent RNA‐Seq studies were performed specifically on cerebral vascular cells. Song et al. 2020 used laser capture microdissection to isolate microvessels from tissue sections prior to RNA extraction and sequencing [344]. As this study was not snRNA‐Seq, it relied on subsequent analysis to delineate cell‐type specific changes [344]. Yang and colleagues used density centrifugation and strainer capture to enrich microvessels from brain tissue, followed by mashing to release cell nuclei for RNA extraction in a technique they refer to as Vessel Isolation and Nuclei Extraction for Sequencing (VINE‐Seq) [345]. Both of these transcriptomic analyses identified novel putative markers enriched in human pericytes, and documented changes in AD and striking differences between expression of genes in human brain tissue and of their homologs in mice [344, 345]. The VINE‐Seq study found that the expression of many risk genes for AD, identified by GWAS, are highly enriched within vascular cell types in humans whereas in mice most of the homologous genes are expressed by microglia [345]. A post‐GWAS analysis of previous snRNA‐Seq data also found GWAS gene expression to be highly enriched in endothelial cells and pericytes in addition to microglia [346, 347]. These studies highlight the pivotal role of vasculature dysfunction in AD.

### 7.3 Novel imaging methods in animal models

The numerous recent advances in the imaging of human brains in AD have been well reviewed [23, 348]. Advances in the imaging of the mouse brain vasculature have yielded detailed whole brain atlases of the cerebral vasculature in experimental models of Aβ and tau accumulation. These include the use of iterative sectioning and imaging of the brains of APP/PS1 mice to provide a 3D reconstruction of cerebral vasculature [165, 349], and the demonstration of reduced hippocampal vascular density in a triple‐transgenic mice (PS1M146V, tauP301L, and APPSwe), by ultrasound measurement of cerebral blood flow [162]. Whole brain imaging using tissue clearing methods such as CLARITY has been shown to be effective [350, 351]. Recently, multiphoton imaging and optogenetic manipulation of mural cells in the live mouse brain was demonstrated as a technique for investigating the role of these cells in regulating rCBF and their dysfunction in the context of AD and cerebral ischaemia [352]. Recently, 2D‐optical imaging spectroscopy (2D‐OIS) was used in a J20‐hAPP mouse model of AD to investigate cerebral haemodynamics while measuring neuronal activity with an inserted electrode [353]. In vivo experimental methods such as these are likely to be increasingly important for mechanistic studies of cerebral vascular abnormalities in AD, and for testing novel therapies.

## 8 CONCLUSION

There is mounting evidence that blood vessels within the brain have altered structure and function from a very early preclinical stage of AD. Structural changes to the larger microvessels (particularly arterioles) include CAA and arteriolosclerosis, but there are also numerous, less immediately obvious, changes to endothelial cells, pericytes and the basement membrane of the microvasculature that reflect the influence of genetic factors, inflammatory mediators, vasoactive peptides, and both direct and indirect endothelial and pericyte responses to Aβ and tau. These structural and physiological alterations to the cerebral vasculature affect vessel maintenance and regeneration, vessel calibre and responsiveness to neuronal metabolic demand, integrity of the BBB, and the metabolism, transport and clearance of many molecules including Aβ. The consequences are a worsening of the reduction and mismatch of brain perfusion in AD, breakdown of the BBB, accumulation of Aβ, parenchymal brain damage, and further damage to the vasculature itself. Transcriptomics of cerebrovascular cells and improved imaging methods of the brain vasculature further highlight the pivotal role of vasculature dysfunction in the development and progression of AD, and the use of the retina as a ‘window into the brain’ may change the way we monitor development and progression of the disease.

## CONFLICT OF INTEREST

We have no conflicts of interest to declare.

## AUTHOR CONTRIBUTIONS

J. Scott Miners devised the concept and scope of the review. Robert A. Fisher wrote the original draft and constructed the tables and figures. J. Scott Miners and Seth Love edited manuscript and provided guidance on content.

## Data Availability

Not applicable.
